# Tackling the Molecular Drug Sensitivity in the Sea Louse *Caligus rogercresseyi* Based on mRNA and lncRNA Interactions

**DOI:** 10.3390/genes11080857

**Published:** 2020-07-27

**Authors:** Gustavo Núñez-Acuña, Constanza Sáez-Vera, Valentina Valenzuela-Muñoz, Diego Valenzuela-Miranda, Gabriel Arriagada, Cristian Gallardo-Escárate

**Affiliations:** 1 Interdisciplinary Center for Aquaculture Research, University of Concepción, Concepción 4070386, Chile; gustavonunez@udec.cl (G.N.-A.); saezvera.c@gmail.com (C.S.-V.); valevalenzuela@udec.cl (V.V.-M.); divalenzuela@udec.cl (D.V.-M.); garriagada@oceanografia.udec.cl (G.A.); 2 Laboratory of Biotechnology and Aquatic Genomics, Center of Biotechnology, University of Concepción, Concepción 4070386, Chile; 3 Instituto de Ciencias Agronómicas y Veterinarias, Universidad de O’Higgins, San Fernando 3070000, Chile

**Keywords:** azamethiphos, deltamethrin, cypermethrin, *Caligus rogercresseyi*, sea louse, lncRNAs, transcriptome

## Abstract

*Caligus rogercresseyi*, commonly known as sea louse, is an ectoparasite copepod that impacts the salmon aquaculture in Chile, causing losses of hundreds of million dollars per year. This pathogen is mainly controlled by immersion baths with delousing drugs, which can lead to resistant traits selection in lice populations. Bioassays are commonly used to assess louse drug sensitivity, but the current procedures may mask relevant molecular responses. This study aimed to discover novel coding genes and non-coding RNAs that could evidence drug sensitivity at the genomic level. Sea lice samples from populations with contrasting sensitivity to delousing drugs were collected. Bioassays using azamethiphos, cypermethrin, and deltamethrin drugs were conducted to evaluate the sensitivity and to collect samples for RNA-sequencing. Transcriptome sequencing was conducted on samples exposed to each drug to evaluate the presence of coding and non-coding RNAs associated with the response of these compounds. The results revealed specific transcriptome patterns in lice exposed to azamethiphos, deltamethrin, and cypermethrin drugs. Enrichment analyses of Gene Ontology terms showed specific biological processes and molecular functions associated with each delousing drug analyzed. Furthermore, novel long non-coding RNAs (lncRNAs) were identified in *C. rogercresseyi* and tightly linked to differentially expressed coding genes. A significant correlation between gene transcription patterns and phenotypic effects was found in lice collected from different salmon farms with contrasting drug treatment efficacies. The significant correlation among gene transcription patterns with the historical background of drug sensitivity suggests novel molecular mechanisms of pharmacological resistance in lice populations.

## 1. Introduction

Undoubtedly, next-generation sequencing technologies (NGS) have become an unprecedented revolution for life sciences, allowing to dramatically increase genomic knowledge in all living species [1,2]. Within this revolution, it is widely evidenced that many non-coding regions of the genome can no longer be considered as “junk” or non-functional segments of the genome because those can have relevant functional and regulatory roles in living cells [3]. Non-coding RNAs have emerged as critical modulators at the molecular level in almost every known cellular function [4]. Long non-coding RNAs (lncRNAs) are a novel class of active RNAs that can influence gene expression of many biological processes in numerous species [5]. Their mode of action is still under debate, but there is a consensus that they are key players at the cellular level [6]. Among the mechanisms involved in gene regulation, lncRNAs modulate epigenetic modifications through recruiting chromatin remodeling complexes, transcriptional control by acting on promoter regions, and post-transcriptional processing by masking *cis*-regulating elements [7]. These diverse mechanisms lead to the involvement of lncRNAs in pivotal biological processes such as cellular development, reproduction, immune response, and metabolism, among many others [8]. Notably, lncRNAs have been associated with mechanisms of pharmacological resistance in arthropod species [9,10].

Although the role of coding and non-coding RNAs in pesticide treatments has been probed in some terrestrial arthropods, there are marine non-model species where the transcriptome responses could be critical for pharmacological resistance monitoring. For instance, the marine copepod *Caligus rogercresseyi* evidences a coding/lncRNAs interaction in response to delousing drugs used in fish aquaculture [11,12]. This species, also commonly named sea louse, causes a high economic impact in the Chilean salmon industry, generating losses of USD $350 million annually [13,14]. Sea louse infections are mainly controlled through pesticide drugs, where the most used in Chile is the organophosphate azamethiphos. Other antiparasitic drugs used in Chile, such as the synthetic pyrethroids deltamethrin and cypermethrin have been reported to be ineffective as a consequence of resistance development [15]. The intensive use of these chemicals leads to the selection of non-susceptible parasites, which in turn can introduce resistant genotypes into wild populations. This phenomenon has been described for *Lepeophtheirus salmonis* [16], which is a louse species that impacts farmed and wild salmon populations in the Northern hemisphere [17]. Azamethiphos drug targets the acetylcholinesterase protein to disrupt synaptic activity [18]. Deltamethrin and cypermethrin are pyrethroids that target key voltage-dependent channels of synaptic pathways to block neuronal excitability mechanisms [19]. Specific resistance mechanisms against pesticides have been described in *L. salmonis,* where a punctual Phe362Tyr mutation in the *acetylcholinesterase* gene and the maternal inheritance of mitochondrial genes confer resistance to azamethiphos [20] and deltamethrin [21], respectively. Meanwhile, in the Chilean species *C. rogercresseyi*, these molecular mechanisms have not been described so far. However, genes and pathways are activated by deltamethrin exposure on *C. rogercresseyi* [22], revealing several genes involved in the response to this drug such as antioxidant genes [23], and the *P-glycoprotein* [24] and *cuticle-protein* genes [25]. Concerning the organophosphate azamethiphos, *C. rogercresseyi* also displays complex transcriptome responses [26], including the *ATP-binding cassette transporters* gene family [27]. 

There are no precise resistance mechanisms described in *C. rogercresseyi* against pesticide drugs, but still tolerant phenotypes to these treatments have been previously described [28,29,30]. The monitoring of these phenotypes in Chile is usually conducted through standard bioassays using the SEARCH Handbook guidelines [31]. This method consists of a visual examination of lice exposed to several drug concentrations, whereby the effective median concentration (EC_50_) is calculated [32]. However, due to the requirement of a large number of individuals and difficulties in the implementation of the bioassay (particularly in field experiments), molecular tools have emerged to complement and evaluate the sensitivity to pesticides [33]. Nonetheless, as the pharmacological resistance mechanisms for *C. rogercresseyi* are not completely elucidated yet, further research is needed for the discovery of novel biomarkers. This study aimed to identify molecular signatures associated with drug resistance based on the interaction between coding/non-coding RNAs. The sensitivity of sea lice populations to these drugs was evaluated through the standard SEARCH bioassays. To validate the novel RNA biomarkers, resistant and susceptible lice strains for three delousing drugs were exposed in parallel to azamethiphos, cypermethrin, and deltamethrin. Notably, specific transcripts were modulated in response to pesticides and the sensitivity level to delousing drugs. In summary, the current study reports novel molecular signatures based on coding/non-coding RNA interactions. This knowledge can be applied as molecular markers to assess and manage the louse drug sensitivity, improving sustainable practices in the salmon industry.

## 2. Materials and Methods 

### 2.1. Lice Samples and Culture in Laboratory-Controlled Conditions

Alive adult *C. rogercresseyi* female parasites were obtained for salmon farms with contrasting treatment efficacy for delousing drugs currently used in Chile: azamethiphos, deltamethrin, and cypermethrin. Treatment efficacy information was provided by the Chilean salmon farming association (SalmonChile, Puerto Montt, Chile). 

These populations came from farms with a contrasting history of drug treatment failures. Low sensitivity populations (putative resistance populations) were classified as those with at least 80% of treatments in the last year below the 60% of efficacies. High sensitivity lice populations (susceptible populations) associated with fish farms with at least 70% of treatments in the last year with efficacies greater or equal than 90% for azamethiphos and 80% for deltamethrin and cypermethrin. A total of six salmon farms were selected, with contrasting efficacy to the three mentioned delousing drugs (Table 1). The parasites were collected from different fish and maintained in seawater at 12 °C with constant aeration for transportation to the Reference Laboratory for Caligidosis (Marine Biology Station, University of Concepcion, Dichato, Región del Bío-Bío, Chile). In the laboratory, gravid adult lice females from each population were maintained in 1 L glass flasks containing filtered seawater with salinity at 32 PSU and temperature at 12 °C. After females hatched, obtained lice at the nauplii-I stage were collected daily until reaching the copepodid stage. Copepodids obtained from different populations were used to infect Atlantic salmon *Salmo salar* in separated 250 L experimental tanks for each louse population. The initial parasitic load for infections was 35 copepodids per fish, and fish were maintained at 12 °C, 32PSU, and constant aeration. Twenty-five days after the infection, adult female and male lice were collected for bioassays with the corresponding delousing drugs.

### 2.2. Bioassays for Transcriptome Sequencing

In the laboratory, six bioassays (two for each drug, one for each population) were conducted following the protocol by the SEARCH Handbook Guideline [31]. Standard bioassays were conducted to evaluate the sensitivity of each population/drug, using azamethiphos (Byelice_^®^_, Bayer Cono Sur, Santiago, Chile), deltamethrin (AMX^®^, Pharmaq South America, Santiago, Chile), and cypermethrin (Betamax^®^, Novartis Chile S.A., Santiago, Chile) drugs. Briefly, the azamethiphos bioassay consisted of the exposure of three groups of 10 parasites (5 females and 5 males) to eight concentrations of the drug: 0 (untreated control), 1, 3, 8, 10, 30, 100, 200, 300 ppb. Sea lice were exposed to azamethiphos for 30 min, and three replicates were considered for each drug concentration. Deltamethrin bioassay consisted of groups formed by the same number of parasites and replicates but exposed for 40 min to different concentrations: 0, 0.5, 1, 2, 3, 9, 20, 40 ppb. Cypermethrin bioassay consisted of the same number of replicates and groups but exposing animals for 30 min to 0, 0.5, 1.5, 5, 15, 20, 50 ppb of the drug. These concentrations were selected based on recommendations by manufacturers and because of previous experiences. After bioassays were finished, Petri dishes containing the parasites and drugs were washed, and animals were maintained for 24 h in seawater in an incubator at 12 °C. Then, the affected condition of the parasites was assigned according to the SEARCH protocol [31]. An affected parasite by the application of the corresponding drug during the bioassay corresponded to sea lice with altered behavior with respect to control parasites. This altered behavior included the inability to attach to the petri dish, circular swimming, and erratic movements. Median effective concentration (EC_50_) was calculated for each drug considering all the concentrations and experimental groups. Parasites were then collected and fixed in the RNALater solution (Ambion^®^, Thermo Fisher Scientific^TM^, Waltham, MA, USA) for RNA-sequencing analyses.

### 2.3. High-Throughput Sequencing from Lice Exposed to Delousing Drugs

Female lice samples were collected from each bioassay and population selecting one concentration per drug: 8 ppb of azamethiphos, 3 ppb of deltamethrin, and 5 ppb of cypermethrin using the mentioned Trizol^®^ reagent method (Invitrogen, Thermo Fisher Scientific^TM^, Waltham, MA, USA). The criteria for selecting these drug concentrations are based on the EC_50_ values obtained after performing bioassays (Table 1). Female lice were used for RNA extraction, including a non-exposed group that was used for each population as a control. All the samples were used for RNA-sequencing following a previously described protocol for *C. rogercresseyi* [34]. Total RNA was extracted from 10 lice for each group using the Ribopure^TM^ kit method (Ambion^®^, Thermo Fisher Scientific^TM^, Waltham, MA, USA). The RNA integrity number (RIN) number of each extracted RNA was calculated for the quality assessment using the TapeStation 2200 instrument (Agilent Technologies Inc., Santa Clara, CA, USA). Only samples with RIN > 8 were selected for further analyses. Good-quality RNA from five selected lice was selected and pooled for libraries construction. From 2 μg of total RNA, dscDNA libraries were constructed using the TruSeq Total RNA kit (Illumina^®^, San Diego, CA, USA). Illumina sequencing was conducted for each library in a MiSeq sequencer (Illumina^®^, San Diego, CA, USA) using a 2 × 250 bp paired-end reads scheme at the Laboratory of Biotechnology and Aquatic Genomics, Interdisciplinary Center for Aquaculture Research, Concepción, Chile. Three sequencing replicates were conducted for each library.

### 2.4. Transcriptome Analyses of Coding Genes in *C. rogercresseyi*

Sequences were trimmed by quality and removing the adapters using the CLC Genomics Workbench software (version 12, Qiagen Bioinformatics). Trimmed reads were de novo assembled in the same software using the following parameters: mismatch cost = 2, insert and deletion costs = 3, contig length > 200 bp, similarity = 0.9, length fraction = 0.8, automatic bubble and word sizes, and contig update = yes. The obtained contigs were mapped to the genome of the host species (*Salmo salar*) and to bacterial genomes to discard sequences contamination. A total of 63,444 contigs were obtained for the sea lice, which were used as a reference for in silico gene expression analyses. RNA-seq analyses were conducted in the same software to calculate gene expression of each dataset using the same settings for costs and similarity fraction as for the assembly. Transcript per million (TPM) values were considered the unit for gene expression analyses. Statistical comparisons among TPM values by the group were obtained by calculating the fold change against the control group using a multi-factorial statistic based on a negative binomial GLM implemented in CLC Genomics software. Contigs having fold change values > |4| and FDR *P*-value < 0.01 were considered as differentially expressed and were extracted for gene annotation. Differentially expressed contigs (in any group against the control) were blasted against SwissProt database [35] by BlastX considering expect value = 10, word size = 11, match/mismatch = 2/−3, and gap costs = 5 (existence)/2 (extension). All the sequences with *E*-value < 1.0 × 10^−6^ were considered as correctly identified in the protein database. The Blas2GO plugin [36] in the CLC Genomics software was used for annotation using the Gene Ontology criteria (GO) into Biological Processes (BP) and Molecular Functions (MF) hierarchies. GO terms enrichment analyses were conducted by Fischer’s Exact Test conducted specific enrichment analyses of GO terms by each library in the Blast2GO plugin by default parameters. In parallel, the transcripts belonging to the Kyoto Encyclopedia of Genes and Genomes (KEGG) pathway of glutamatergic synapse of arthropods were downloaded and used as a reference for RNA-seq analyses of each sequenced library calculating the TPM values by the same specified parameters. 

### 2.5. LncRNAs Identification and Characterization from Lice Transcriptome

Long non-coding RNAs (lncRNAs) were identified from sea lice transcriptomes using a previously described method for *C. rogercresseyi* [11]. De novo assembly previously obtained was used for lncRNAs identification, discarding all the contigs with average coverage < 50 reads. Open reading frames (ORF) were identified in these contigs, and all of those having ORFs longer than > 200 bp were also discarded. The coding potential of the remaining sequences was evaluated using Coding Potential Assessment Tool (CPAT) [37] and Coding Potential Calculator (CPC) [38] software, discarding all the contigs with coding potential. A BlastX against the whole non-redundant databases from Genbank (National Center for Biotechnology Information), EMBL (European Laboratory of Molecular Biology), and DDBJ (DNA Data Bank of Japan) was used to discard positive hits with known proteins. The CD-search tool from NCBI’s was also used to discard those sequences having positive hits with known coding domains. The final filter consisted of a nucleotide-blast (BlastN) analysis to discard those having positive hits with known coding genes. The remaining sequences were considered putative lncRNAs in this sea lice transcriptome. 

Characterization of lncRNAs consisted in comparison of known features of lncRNAs such as length, GC content, and total gene expression with coding genes. This is because it was described that lncRNAs have lower length, %GC, and expression than mRNA when they are obtained from the same transcriptomes [11]. Gene expression analyses of putative lncRNAs were conducted applying the same method as for coding genes, including TPM calculation and statistical analyses. To infer the putative interactions of lncRNAs with coding genes, a correlation analysis of gene expression was conducted. Here, Pearson’s correlation coefficient (r) among all the differentially expressed coding and lncRNAs genes were estimated. This calculation was conducted in R software [39] and was visualized by plots made in the Corrplot package [40]. Correlations between the transcription expression of a lncRNA and a coding gene were statistically significant if the *r* value was > 0.9 and FDR *P*-value < 0.01.

### 2.6. Validation of Coding Genes and lncRNAs Transcription Levels

A group of the top-20 most differentially expressed coding genes were selected according to their correlation values. Primers for qPCR were designed for all of these selected transcripts. In parallel, RNA obtained for RNA-seq analyses were used for quantitative-PCR reactions, applying the ΔΔ_CT_ method [41]. In this case, RNA extraction from male samples, obtained using the same methodology explained for females were also included. PCR reactions were conducted using the kit Maxima SYBR Green/ROX qPCR Master Mix (Thermo Fisher Scientific^TM^, Waltham, MA, USA) and the b-tubulin genes as endogenous controls since it was previously validated for drug response in *C. rogercresseyi* [26]. Primer efficiency was checked by the dynamic range of five serial dilutions to achieve 90–110% of amplification efficiency. Principal component analyses (PCA) were conducted to evaluate the association of gene transcription patterns for mRNA and lncRNA with sexes and strains. Pearson’s correlations coefficients were calculated between gene transcription values and the number of affected animals. Statistical significances and *r* values were calculated as explained above. Relative expression changes of significantly correlated genes for each drug (azamethiphos and deltamethrin) were compared with the visual examination (e.g., number of affected animals) at each concentration used in the bioassay.

## 3. Results

### 3.1. Coding Transcriptomes Associated with Drug Exposure

**Figure 1:**
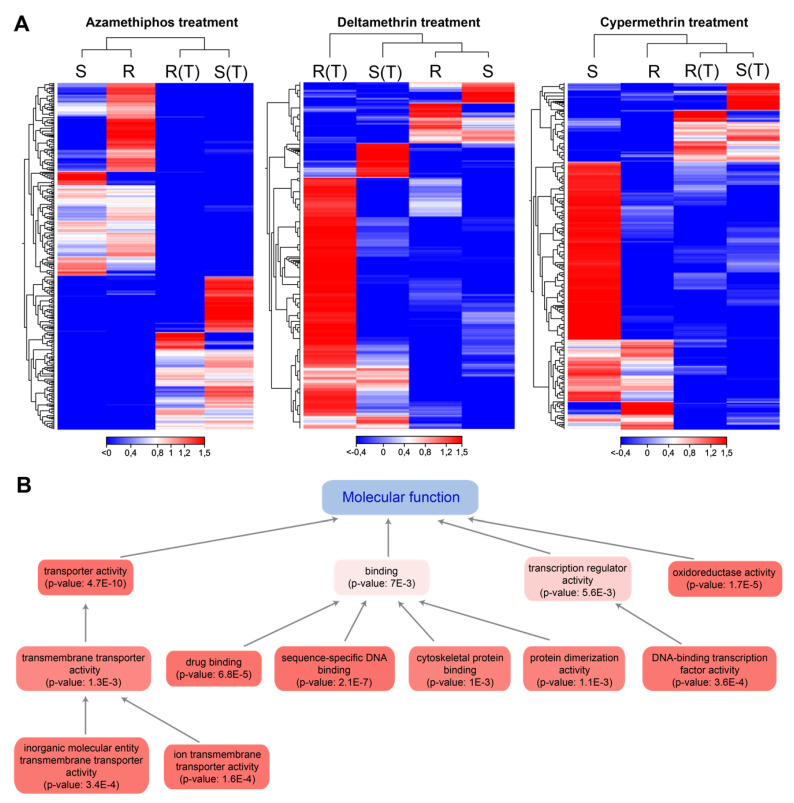
Transcriptome patterns of coding genes of sea lice exposed to pesticide drugs. (**A**): heatmap based on TPM(Transcript per million) calculation and hierarchical clustering on Manhattan distances with average linkage. Double clustering was applied including features (genes) and samples (strains and treated/untreated condition). Clustering on gene expression levels are indicated in the left of each heatmap, and on samples on top of each heatmap. Red colors mean upregulated coding genes, blue colors downregulated genes. R: resistant population to a specific drug, S: susceptible populations for the corresponding drug; (T) treated lice (exposed to the corresponding drug). (**B**): Enrichment of GO (Gene Ontology criteria) terms related to molecular functions in all the combined datasets (treated vs. untreated parasites for the three drugs). Enrichment analyses were based on p-values obtained by Fischer’s Exact Test. Red colors and mean significantly enriched GO terms.

Sea lice samples for RNA sequencing were collected through bioassays with azamethiphos, deltamethrin, and cypermethrin drugs. Then, de novo assembly of cDNA libraries from treated and control parasites yielded 63,444 high-quality contigs (N50 = 1156 bp average length and average coverage > 50 reads). RNA-seq analyses yielded a total of 1,717 significant differentially expressed genes of sea lice exposed to drugs with respect to control non-treated groups (|fold change| > 4 and FDR *P*-value < 0.01). These transcripts exhibited specific expression patterns associated with each drug in the affected individuals, displaying an upregulated gene cluster after azamethiphos, deltamethrin, and cypermethrin treatments, respectively. These patterns were also associated with the lice populations, evidencing expression differences between resistant and susceptible strains for each drug (Figure 1A). There was also a cluster of downregulated genes for each drug in affected sea lice exposed to drugs with respect to unaffected parasites. From the cluster of up-regulated genes for azamethiphos exposure in affected parasites, several cellular structure genes were found such as actin and myosin chains, but also other genes related to synapse transduction (e.g., ATB binding cassette), detoxication process (e.g., peroxinectin, trypsin-1-like, metalloproteinases), and cuticle formation genes were present in this group. Clusters for genes associated with affected lice after cypermethrin treatment were less abundant, but corresponded to a diverse amount of biological processes, and included detoxication genes such as cytochrome p-450 and metalloproteinases. Deltamethrin exposure comprised a cluster of many upregulated genes in affected parasites related to cellular structural (nuclear and mitochondrial factors) functions and DNA replication enzymes. A full gene list of activated genes by cluster can be found in Table S1.

BlastX analysis of differentially expressed transcripts yielded 761 coding genes that were correctly annotated against the SwissProt database (2020_03 release) with an *E*-value < 1.0 × 10^−6^ cutoff. Annotation by Gene Ontology (GO) showed that 952 GO terms were found for biological processes, 300 for molecular functions, and 222 for cellular components. Regarding molecular functions, enrichment analyses of GO terms from all the differentially expressed genes exhibited that the most abundant terms were related to binding (particularly drug binding and sequence-specific DNA binding), transporter activity (e.g., transmembrane transporters), regulation of transcription and oxidoreductase activity (Figure 1B).

**Figure 2:**
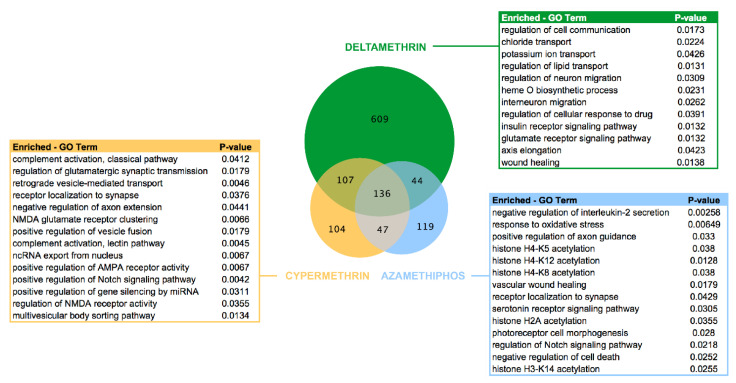
Enrichment of GO terms analyses by each drug. Venn diagram shows all the differentially expressed and annotated coding genes in sea lice exposed to each drug with respect to the control group. Enrichment analyses were conducted based on Fischer’s Exact Test comparing GO terms for each drug using GO terms for the control group as a reference. The green table shows the top significant enriched GO terms for deltamethrin exposure, yellow for cypermethrin, and blue for the azamethiphos drug. Considered GO terms were those included in biological processes and molecular function categories.

Differentially expressed genes (DEGs) were mainly modulated in lice exposed to deltamethrin. From 896 transcripts highly regulated, 609 DEGs were exclusives genes in response to this pyrethroid. Notably, GO annotations were related to nervous system categories such as interneuron migration, ion transport, axis elongation, and glutamatergic synapse (Figure 2). Furthermore, 394 genes were differentially expressed after cypermethrin treatment, where 104 DEGs were exclusively regulated. Enriched GO analysis revealed the association of cypermethrin with nervous system categories, and also with complement pathway activation, ncRNA export from the nucleus, and regulation of gene expression by miRNAs. Azamethiphos exposure triggered the regulation of 346 significant DEGs, including 119 exclusives related to interleukin-2 secretion, oxidative stress, and several related to epigenetic regulation as histone modifications.

### 3.2. Glutamatergic Synapse Pathway

**Figure 3:**
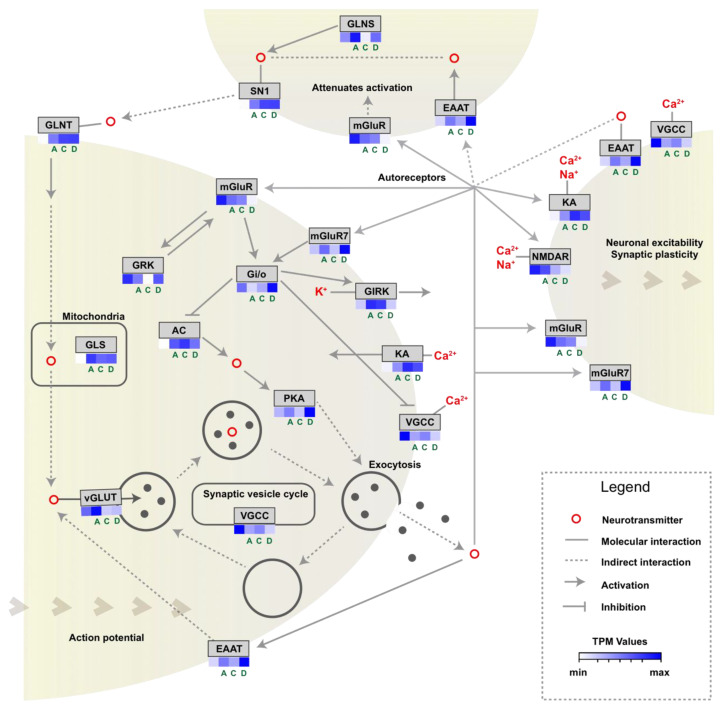
Schematic representation of the glutamatergic synapse pathway and expression levels of sea lice exposed to three drugs. A: azamethiphos, C: cypermethrin, D: deltamethrin, unlabeled squares: untreated samples. Color gradients mean the TPM values of each group, where blue colors are higher levels.

The transcriptome analysis evidenced a high number of DEGs related to the glutamatergic synapse pathway in response to drug treatments. Consequently, a more in-depth analysis to determine the molecular components of this pathway was conducted. Most of the transcripts involving the KEGG pathway for glutamatergic synapse were found at the transcriptome level. Most of these genes were upregulated after drug exposure, including key genes such as glutamate-ammonia ligase (GLNT), glutaminase-2 (GLS), ionotropic kainate receptors (KA), metabotropic glutamate receptor-7 (mGluR7), potassium voltage-gated channel subfamily J (GIRK), protein kinase A (PKA), adenylate cyclase-1 (AC), solute carrier family-1 (EAAT), and family-38 (SN1). Other negative regulators of neuronal excitability were downregulated such as the other metabotropic receptors members (mGluR), G protein-coupled receptor kinase-2 (GRK), and G protein subunit β (Gi/o), which were downregulated after azamethiphos and cypermethrin exposure, but upregulated after deltamethrin treatment (Figure 3).

### 3.3. LncRNA Characterization and Expression Patterns

**Figure 4:**
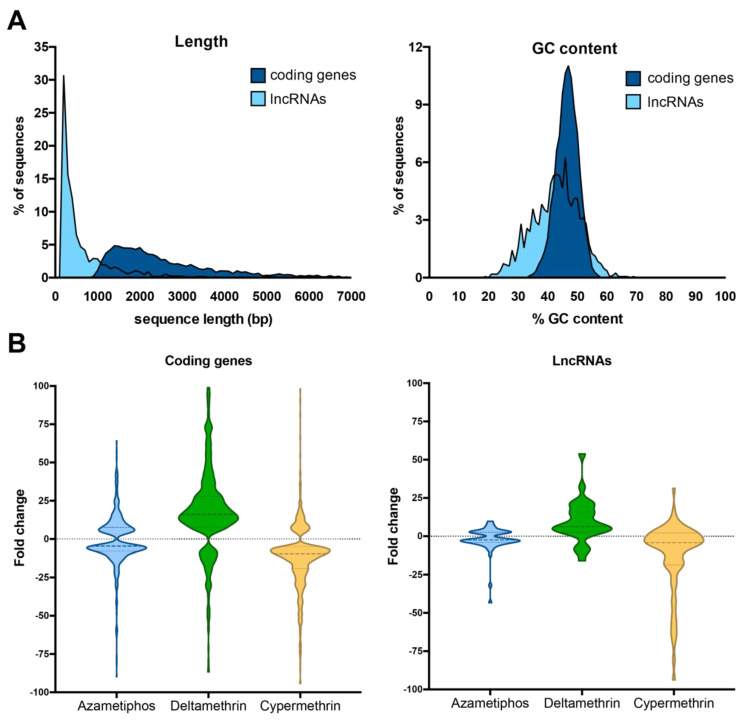
Characterization of lncRNAs in the transcriptome of sea lice exposed to pesticide drugs. (**A**)-left: histogram of sequence length (bp) of coding genes (blue) and lncRNAs (light blue); (**A**)-right: histogram of %GC content of coding genes and lncRNAs. (**B**): Violin plots showing the total gene expression of coding genes and lncRNAs by each treatment (considering both populations per drug). Gene expression is calculated by fold change of TPM values of each group to the control group.

Long non-coding RNAs (lncRNAs) were also characterized in the transcriptomes of sea lice exposed to delousing drugs. A total of putative 1676 lncRNAs were found in the sea lice transcriptomes. LncRNAs were shorter and had lower GC content than coding genes (Figure 4A). Total expression levels expressed as fold-change values against the control group were also lower in lncRNAs to coding genes (Figure 4B).

**Figure 5:**
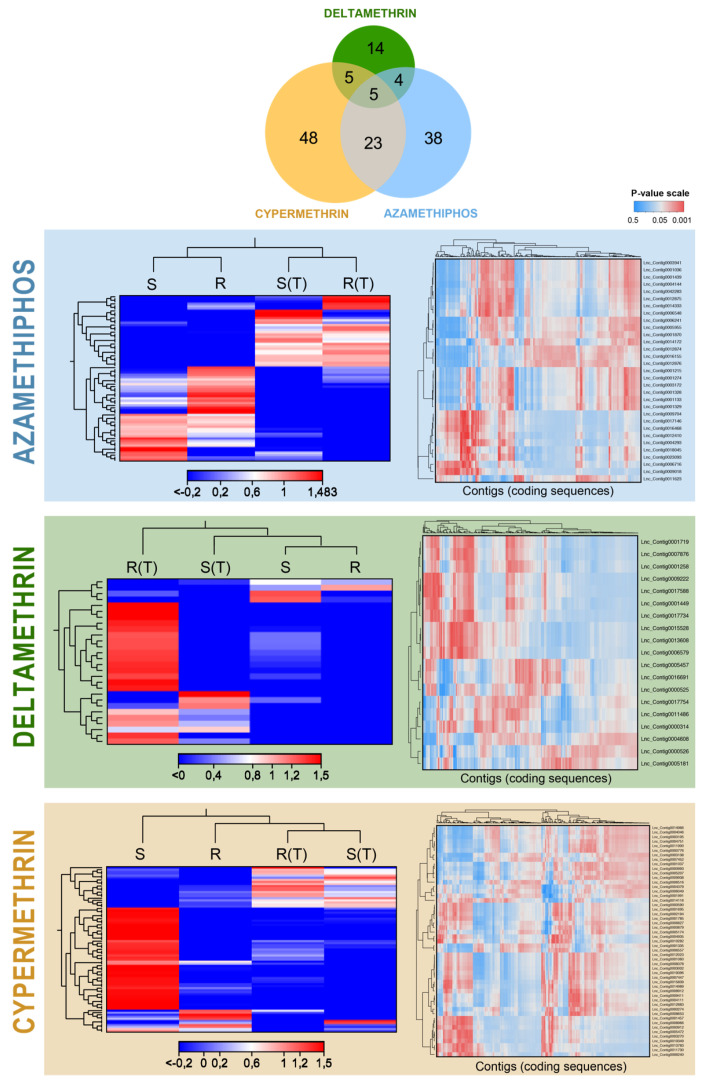
Expression patterns of lncRNAs in sea lice exposed to each drug. Left panels: heatmap of TPM values for lncRNAs clustered by Manhattan’s distances with average linkage. The Venn diagram in the center shows the differentially expressed lncRNAs by a drug (fold change > |4| and FDR *P*-values < 0.01). Right panels: the p-values matrix obtained from the correlation of lncRNAs’ TPM values with expression from coding genes. In the *p*-value matrix, red means significant correlations between the TPM value of a lncRNA and a coding sequence. R: resistant population to a specific drug, S: susceptible populations for the corresponding drug; (T) treated lice (exposed to the corresponding drug).

A total of 137 differentially expressed lncRNAs were found in lice exposed to delousing drug treatments (|FC| > 4 and FDR *P*-value < 0.01). Two clear clusters of transcript expression levels were found for each drug according to both populations. For each pesticide, there is one cluster of differentially expressed lncRNAs that is related to the bioassay (treated samples versus non-exposed parasites). There is another cluster of differentially expressed lncRNAs related to the population, where differences in expression trends were found with respect to the resistance or susceptibility of the experimental groups (Figure 5). Most of these lncRNAs, 81 in total, were differentially expressed in response to cypermethrin exposure, where 48 transcripts were exclusives for this delousing drug. Azamethiphos exposure triggered significant expression changes in 70 lncRNAs, and 38 of them were exclusives for the organophosphate. Sea lice exposed to deltamethrin showed 28 differentially expressed lncRNAs against the control groups, being 14 exclusives for this pesticide. Various of these differentially expressed lncRNAs were significantly correlated (|Pearson’s correlation| > 0.9 and FDR *P*-value < 0.01) to differentially expressed coding genes, finding 233 significant correlations for cypermethrin, 196 for azamethiphos and 89 for deltamethrin treatment (Figure 5).

### 3.4. Validation of Specific Transcripts Associated with Delousing Drug Tolerance/Susceptibility

**Figure 6:**
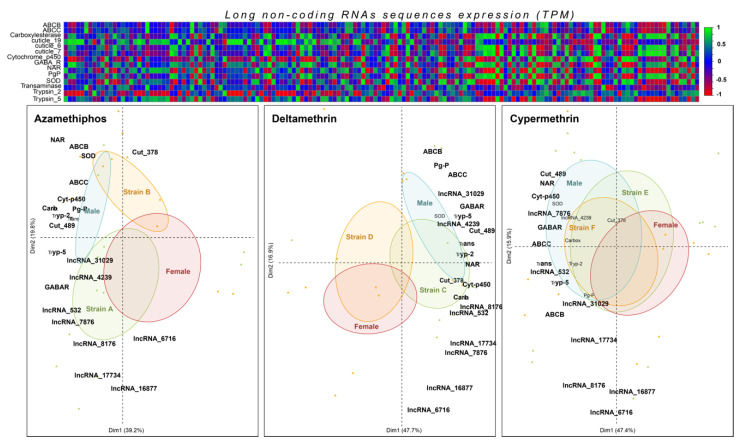
Correlation between selected candidate lncRNAs and coding genes by RNA-seq and qPCR analyses. In the top is shown a correlation matrix (correlation coefficients) among TPM values of selected candidate genes and lncRNAs (top-differentially expressed RNAs) obtained by RNA-seq. Green and red colors mean significant correlations (positive or negative significant correlations, respectively). At the bottom, a principal component analysis (PCA) is shown by each drug. PCA was constructed based on the relative expression values obtained by ΔΔ_CT_ method of coding and lncRNAs genes. The dependent variable was expression values, while the independent variable was each sample corresponding to sea lice of two populations exposed to the corresponding drug. Sexes and strains were considered an independent factor for grouping gene expression values. A, C, and E represent resistant strains for the corresponding drugs, while B, D, and F to susceptible strains.

From the previous analyses for coding and lncRNAs mining with the response to delousing drugs, a subset of 21 top-differentially expressed transcripts were selected for validation and testing in bioassays conducted through different lice strains. Principal component analyses (PCA) showed that the three pesticides could be associated with specific RNA markers. Notably, there was a clear relationship between the transcription activity measured in females and males exposed to delousing drugs. This finding is more marked in azamethiphos and deltamethrin treatments (Figure 6). As for the comparison between lice strains, it was possible to evidence a clear pattern in azamethiphos and deltamethrin treatments according to the respective resistant/susceptible populations.

In contrast, cypermethrin treatment did not exhibit differentiation by specific gene transcription profiles. It was possible to observe that most differentially expressed transcripts of resistant lice strain to azamethiphos corresponded to lncRNAs, while the other population revealed a correlation with genes such as cuticle protein 378, super-oxide dismutase, ABCB and ABCC transporters. On the other hand, most of the selected transcripts were almost exclusively associated with the deltamethrin-resistant strain, while there was no transcription associated with the susceptible strain (Figure 6).

**Figure 7:**
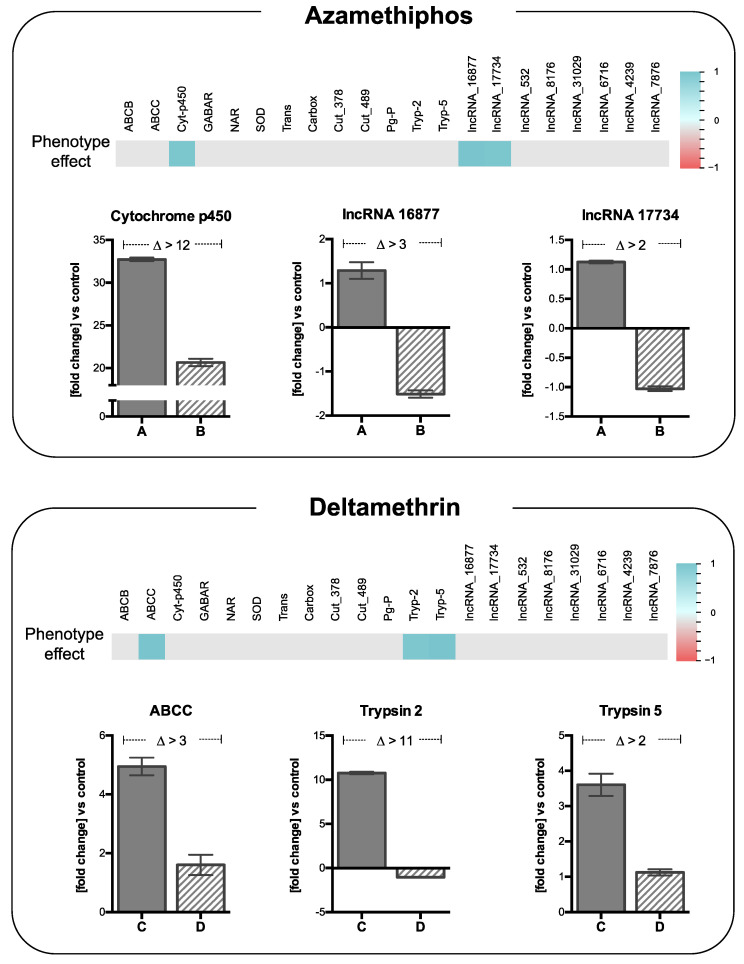
Correlation among gene expression of significantly expressed mRNAs and lncRNAs with phenotype effect of bioassays. At the top of each panel is the correlation value among the phenotype effect (number of affected sea lice by the corresponding bioassay) and the expression patterns of selected coding and non-coding genes. The number of affected lice was obtained throughout the bioassays by each drug concentration used for the three drugs. Column bars represent the means for the fold-change differences based on the relative expression of treated female parasites against untreated parasites for each drug at the corresponding concentrations. Selected genes corresponded to those with significant correlation (|correlation coefficient| >0.9) between coding genes and lncRNAs in both resistant and susceptible strains. Error bars correspond to standard deviations (*n* = 3 parasites). A: resistant population to azamethiphos, B: susceptible population to azamethiphos, C: resistant population to deltamethrin, D: susceptible population to deltamethrin.

Significant correlations (*r* > |0.9| and FDR *P*-value < 0.01) were found between the results of the bioassays and the expression patterns of three genes for azamethiphos and deltamethrin treatments (Figure 7). For the azamethiphos bioassay, *the cytochrome p450* and the *lncRNAs 16877 and 17734* were significantly correlated to the number of affected animals. Here, 2 to >12 differences in the fold-changes against non-treated lice between strains have been found. For deltamethrin, the expression pattern of *ABCC* and *trypsin 2* and *5* genes were significantly correlated to the bioassay, evidencing fold-changes differences from 2 to >11 in both strains.

## 4. Discussion

Functional genomics analysis based on high-throughput sequencing techniques represents one of the most valuable tools to uncover key molecular responses in marine organisms. The present study highlighted the complexity of the transcriptome responses of lice exposed to pesticides. Herein, most of the differentially expressed genes in lice treated with pesticide drugs were associated with the parasite′s nervous system (Figure 1 and Figure 2). This feature was expected due that the tested pesticides target specific proteins (acetylcholinesterase, voltage-dependent channels), impacting the louse motility and consequently its survival. However, cumulative pieces of evidence demonstrate that these drugs trigger global gene responses in treated animals, which are not merely associated with target genes/proteins [19,22,26,42,43,44,45]. Indeed, the results of this study also evidenced complex gene responses in *C. rogercresseyi* exposed to the mentioned pesticides. Notably, clear differences in the most significantly enriched biological processes and molecular functions as well as its specific association with delousing drugs were observed (Figure 2). Deltamethrin gene responses were mainly associated with nervous system processes, but not restricted to one particular synaptic pathway, also finding “interneuron migration” and “axis elongation” as enriched processes. This response was in some way expected because deltamethrin is known for disrupting synaptic activity in many levels [46] and because its molecular targets are key components in many different synaptic pathways [47]. Conversely, to what was expected, lice treated with cypermethrin showed novel enriched processes such as those involved in specific synaptic receptors (AMPA, NMDA, Notch), but also those in “regulation of gene expression by miRNA” and ncRNAs action. Fewer studies are explaining the molecular mechanisms associated with cypermethrin action, but our results confirm its implication in the parasite′s nervous system. Notably, the receptors highly modulated by cypermethrin are involved in general neurotransmission systems and also in memory formation [48,49,50,51]. Concerning Notch receptors, previous studies have evidenced its transcriptome modulation in lice exposed to azamethiphos and deltamethrin drugs [52]. The present study confirms that cypermethrin drug could also disrupt Notch signaling, which is involved in neuronal function and development. Furthermore, this is the first time that pathways related to the ncRNAs processing are being involved in cypermethrin exposure in a marine species. However, this was also confirmed in the other analyses from this study where many lncRNAs were activated/inhibited by the action of this drug. In relation to azamethiphos exposure, other pathways related to the synaptic activity of sea lice were enriched such as “regulation of axon guidance,” which is critical for neuronal processing [53], and the mentioned Notch signaling system. Oxidative stress was also a significantly enriched process, which is expected by previous studies on sea lice exposed to this delousing drug [23]. According to our knowledge, a significant finding was the transcriptome modulation of molecular functions related to histone modifications (e.g., acetylation of different histone types) in lice exposed to azamethiphos. This suggests that novel epigenetic regulation mechanisms may be involved with sea lice in the response to this organophosphate pesticide. Epigenetic regulations were found in other organisms exposed to a similar organophosphate molecule (chlorpyriphos) [54]. Indeed, pharmacological resistance induced by epigenetic modification mechanisms has been studied in model species, where epigenetic may lead to the development of transcriptional memory and then to changes in key-genes that are critical for drug response [55]. *Cytochrome p450* gene regulation by histone modifications causes changes in the activation of this gene, which was strongly linked to sea louse response to azamethiphos. This study suggests the reported involvement of these modifications in pharmacological resistance [56]. In Chilean salmon farming, this could be one of the explanations of the faster emergence of tolerance to azamethiphos, which took only a few years since its introduction in 2013, than deltamethrin that was used in the last decade and is still used in various local salmon farms [57]. However, further research in the epigenetic modifications that could induce sea lice resistance to azamethiphos has to be conducted to prove this hypothesis.

The more in-depth analyses conducted on glutamatergic synapse pathway regulation suggest that the three drugs could potentially disrupt the normal functioning of this system (Figure 3). Overall, it was interesting that most of the changes are tending to increase the neuronal excitability of sea lice after drug exposure, increasing the expression levels of activators, and downregulating the inhibitors. This response is common in species exposed to organophosphates derived from the inhibition of cholinesterase proteins, causing an overactivation of synaptic signals, that in turn, produces metabolic and homeostatic imbalances [58,59]. Indeed, this is confirmed by the evident upregulation of *ionotropic glutamate kainate receptors* (*KA*), which are critical factors enhancing neuronal response leading to hyperexcitation [60]. Pyrethroids, such as deltamethrin and cypermethrin are also chemicals compound with demonstrated effect on neuronal hyperexcitability by binding to voltage-dependent proteins and holding those open [46,61]. The patterns obtained in this study are congruent to those evaluated in lice exposed to azamethiphos and deltamethrin in a previous study, observing stochastic changes in this same synaptic pathway [62]. In this study, cypermethrin drug was also included, and the findings were similar regarding glutamatergic synapse upregulation.

In arthropods species, insecticide resistance is commonly described as the emergence and enrichment of resistant alleles at the lice population level [63]. These alleles include those involved in single mutations on pesticides’ target proteins, inhibiting the correct binding or by the action of detoxification mechanisms [64]. These latter mechanisms involve the upregulation of metabolic factors that could detoxify by processing the drug and enhance the action of cytochrome p450 protein degrading deltamethrin drug [65]. Other proteins conferring resistance to deltamethrin by the detoxication process are trypsin in arthropod species [66]. In the salmon louse *Lepeophtheirus salmonis*, punctual mutations in the *sodium channel* gene were found in a deltamethrin-resistance strain [67]. Detoxication mechanisms conferring resistance have also suggested in the same species by the action of monooxygenase genes able to process deltamethrin [68]. Detoxication by trypsin proteases genes was also suggested in the sea louse *C. rogercresseyi* against deltamethrin drug [69]. However, recently, in *L. salmonis* species, another resistance mechanism was described, which is related to mutations and enhance expression levels in genes of the mitochondrial genome in strains resistant against deltamethrin [21,70]. Resistance mechanism against azamethiphos in arthropods has also been suggested by the action of punctual mutation in target acetylcholinesterase (*AChE*) genes or by enhancing detoxication mechanisms [71,72]. In the salmon louse *L. salmonis*, resistance mechanisms against azamethiphos have been mainly linked to a punctual mutation in the *AChE* gene, particularly the non-synonymous Phe362Tyr mutation [20,73,74,75]. However, this mutation was not found in *C. rogercresseyi* sea louse, although mutations and gene variants were found in the same genes but not associated with resistance strains yet [33]. Besides, transcriptomic studies suggest that the resistance mechanisms against azamethiphos could not be restricted to the presence of single mutations on target genes in *C. rogercresseyi*, but also involving many genes and pathways [25,26,27,52,62] and non-coding RNAs [11,76]. The results obtained in this study strongly suggest the hypothesis of global and wide response mechanisms conferring resistance to delousing drugs in *C. rogercresseyi*. These mechanisms are not fully demonstrable yet, because *C. rogercresseyi* resistance strains have to be obtained and characterized at laboratory conditions. The research group involved in this study is currently conducted the whole genome sequencing for *C. rogercresseyi*. Here, the platinum-standard full genome will be pivotal to disentangle the resistance mechanisms at the genomic level and understand how genetic and environmental factors modulate this phenotype. From our point of view, this study contributes with relevant information about the association between coding genes and lncRNAs of lice strains from populations with contrasting efficacies to deltamethrin and azamethiphos. 

Moreover, the present study describes new knowledge of how lncRNAs are involved in the pesticide response, contributing to understanding the complexity of the molecular regulation in sea louse against delousing drugs. This class of non-coding RNAs is critical in the *C. rogercresseyi* lifecycle as are involved in a vast and complex repertoire of the biological process during fish infection [77]. Notably, the current results are congruent with a previous study [11], where the correlation between drug response and genes such as trypsin, myosin, and mitochondrial-related genes were overregulated (Table S2). The present study contributes to the association of some lncRNAs with a specific population of sea lice, specifically those who were less affected by azamethiphos (Figure 6 and Figure 7). The lncRNAs 16877 and 17734 were correlated to cuticle protein and P-glycoprotein (*PgP*) genes respectively, suggesting that these ncRNAs are involved in nervous system transporters and the cuticle formation of the species [24,25]. In contrast to what was expected by the previous biological processes involved in cypermethrin response, there were not lncRNAs found to be explicitly associated with both populations to this pesticide. This could be caused due to the selection of gene/lncRNAs for validation because no coding genes could be associated with these populations (Figure 6). To solve this fact, two well-characterized populations of sea lice with contrasting efficacy of cypermethrin should be used, but this is difficult to obtain those because of the reduced use of this drug nowadays [78].

Monitoring sea lice susceptibility to drugs in Chile is commonly conducted by standard SEARCH bioassay [30,31,32]. One of the critical points of this method is the correct assignation of an affected parasite not overlooking non-affected lice. The discovery of the molecular markers validated in this study could dramatically reduce operational bias by assigning the conditions based on differences in the gene expression of parasites against the control. The *cytochrome p450* and *lncRNAs 16877* and *17734* are good candidates for the assignation of an affected condition in sea lice after azamethiphos bioassays, while *ABCC* transporter and *trypsins 2* and *5* are good candidates for deltamethrin bioassays.

## 5. Conclusions

The results obtained in this study demonstrated that azamethiphos, deltamethrin, and cypermethrin drugs trigger relevant genes related to the nervous system function in the sea louse *C. rogercresseyi*. Nonetheless, the molecular processes that are being activated/inhibited in lice exposed to these pesticides are not restricted to synaptic pathways or specific target proteins. There are many putative key molecular players in response to pesticides that are not fully characterized yet, such as those involved in epigenetic modifications and their interaction with miRNAs/ncRNAs. Notably, lncRNAs were significantly associated with the response of delousing drugs, opening new scientific questions about how lncRNAs can regulate the molecular responses in lice exposed to delousing drugs, and also how is the heritability of ncRNAs involved in drug resistance. Importantly, these biomarkers can be applied to estimate genetic diversity at the population level. Finally, a selected group of coding genes and lncRNAs are proposed as a molecular method to test lice drug sensitivity in Chilean salmon farms.

## Figures and Tables

**Table 1:** Salmon lice strains used for this study. Treatment efficacy corresponds to the mean of the treatment efficacies in the last year from the original populations where female lice were obtained to obtain the strains. Bioassay results correspond to the result of each bioassay in the strains cultivated in the laboratory (F_1_). EC50: effective concentration on 50% of the population, CI: confidence interval.

Strain	Drug	Location	Treatment Efficacy	EC50	95% CI
A	Azamethiphos	Aysén	<60%	13.72	6.392–32.6
B	Azamethiphos	Aysén	>92%	0.4778	0.0765–1.275
C	Deltamethrin	Los Lagos	<50%	3.042	1.826–5.285
D	Deltamethrin	Aysén	>80%	1.424	0.634–3.746
E	Cypermethrin	Los Lagos	<40%	9.709	3.155–57.84
F	Cypermethrin	Los Lagos	>80%	2.178	0.972–5.259

## Supplementary Materials

The following are available online at https://www.mdpi.com/2073-4425/11/8/857/s1, Table S1: BLAST results for differentially expressed genes obtained for specific clusters in heatmaps, Table S2: Correlation values for lncRNA and mRNA expression.
